# Bipolar disorder in late life: clinical characteristics in a sample of older adults admitted for manic episode

**DOI:** 10.1186/1745-0179-4-22

**Published:** 2008-07-29

**Authors:** Alessandra Benedetti, Pietro Scarpellini, Francesco Casamassima, Lorenzo Lattanzi, Maria Liberti, Laura Musetti, Giovanni Battista Cassano

**Affiliations:** 1 Department of Psychiatry, Neurobiology, Pharmacology and Biotechnologies, University of Pisa, Via Roma, 67, Pisa, Italy

## Abstract

**Background:**

Although manic episodes in older adults are not rare, little published data exist on late-life manic episodes. Resistance to treatment and concomitant neurological lesions are frequent correlates of elderly mania. The aim of this study was to investigate the prevalence of hospitalizations due to mania in patients older than 64 years through a period of 5 years in an Italian public psychiatric ward. Moreover, we aimed at describing clinical presentation of elderly manic episodes.

**Methods:**

A retrospective chart review was conducted in order to describe clinical presentation of 20 elderly patients hospitalized for manic episode; moreover, we compared age at onset, the presence of family history for mood disorders, psychosis and irritability between the elderly group and a matched group of 20 younger manic inpatients.

**Results:**

Seven percent of the whole inpatient elderly people suffered from mania. Half of those patients had a mood disorder age at onset after 50 years and 5 patients were at their first manic episode. Geriatric- and adulthood mania showed similar clinical presentation but younger people had more frequently a mood disorders family history.

**Conclusion:**

Half of our older manic inpatients consisted of "classic" bipolar patients with an extension of clinical manifestations into later life; the other half of our sample was heterogeneous, even though it was not possible to identify clearly which patients may have had vascular lesions related to the onset of mania.

## Background

Mania has been estimated to represent the cause of 4.6% to 18.5% of all geriatric psychiatric admissions [1,2] and 10% of new-onset mania cases have been found to occur in individuals over the 50 years [3]. Studies comparing mania in older and younger adults have found no substantial differences in clinical presentation. It has been suggested, however, that mania in older patients is less severe and manifests with more irritability, confusion, psychosis, and mixed features [4].

Age at onset, another topic of investigation in research conducted on bipolar disorder (BD) in older people, has been proposed as representing a specifier for more homogeneous groups of BD families, facilitating the mapping of susceptibility genes thereby [5]. In this line, several findings from literature reported higher rates of family history in younger bipolar people [6].

The present retrospective chart-review study was aimed at investigating the prevalence of hospitalizations due to mania according to DSM-IV [7] in the total geriatric population admitted to the University of Pisa's Psychiatric Department wards between August 1998 and August 2003. We also aimed at describing clinical features of older manic patients and finally, we compared age at onset, presence of family history for mood disorders, psychosis and irritability in the older patients sample and in a sample of manic inpatients under the age of 65 years.

## Methods

This retrospective chart-review study was conducted at the University of Pisa's Psychiatric Department wards during the period of August 1998-August 2003. The study protocol was duly approved by the local ethical committee.

During the sampling period, 284 patients older than the age of 64 years had been admitted. Twenty of these participants (11 women and 9 men) were admitted because of mania and their medical files were therefore reviewed and included in the analyses. We then randomly selected a sex-matched control sample of 20 inpatients with a diagnosis of mania from all patients under the age of 65 years who had been admitted to the wards at some point during the study period.

At the moment of admission each patient is routinely clinically examined in order to exclude a diagnosis of dementia or any other organic mental disorders. Demographic, clinical features and pharmacological treatment at discharge of the participants were recorded in a specific form by a resident (PS) in psychiatry from the University of Pisa's Department of Psychiatry, who had clinical experience with patients suffering from mood disorders and who had undergone a specific training. Neuroradiological exams performed during the hospitalization were also examined, when available. Age at onset, presence of family history for mood disorders, psychosis and irritability were compared in elderly and young patients.

### Statistical Analyses

Continuous data were compared using either the Student's T-test. Pearson's chi-square was used to compare contingency table proportions, and the two-tailed Fisher's Exact Test (FET) was used to analyze contingency tables with less than five observations per cell. All statistical analyses were performed with the statistical package Intercooled Stata, 8.2.

## Results

Seven percent (7%) of all inpatients above 64 years of age had been discharged with a diagnosis of mania according to DSM-IV criteria. Mean length of hospitalization was 17.3 (SD 20.1) days. The mean age of elderly patients was 74 years (± 6.1). Nine patients were men; 14 were married; 4 were widowed or widowered; and 2 were single. Most patients lived with their families, but two patients were institutionalized.

Table 1 reports data about the 20 subjects. The mean mood disorder onset age of patients in our sample was 48.9 (SD 23.6). Age at onset was after 64 in seven patients. Five patients were at their first manic episode; of them, three (pt 2, 3, 17) had had depressive episodes in the past and in two patients (pt 16, 19), aged 79 and 88, respectively, current mania represented the onset of mood disorder. More than half of patients (N = 12) displayed irritability, and psychotic symptoms (N = 15) during current episode, and only 6 patients reported at least one first-degree relative with mood disorders (two missing values).

**Table 1:** Socio-demographic characteristics of 20 elderly inpatients admitted for manic episode.

Pt	Gender	Age	Age at onset	Polarity of onset	First episode	First mania	Irritability	Hallucinations or delusions	Mood disorders family history	General Medical Conditions	Neuroimaging
1	F	71	39	Mix	No	No	Yes	Yes	Yes	Hypertension	-
2	F	75	37	Dep	No	Yes	No	No	Yes	-	-
3	F	81	17	Dep	No	Yes	No	No	Yes	-	Cortical atrophy
4	F	69	18	Dep	No	No	No	Yes	Yes	-	-
5	F	76	?	Dep	No	No	No	Yes	No	Infection	-
6	F	80	75	Man	No	No	Yes	Yes	?	-	-
7	F	77	67	Mix	No	No	No	Yes	?	Hypertension	Chronic vascular ischemia
8	F	80	79	Man	No	No	No	Yes	No	Hypertension	Lacunar infarct
9	M	67	60	Dep	No	No	Yes	Yes	No	Hypertension	-
10	M	74	41	Mix	No	No	Yes	Yes	Yes	Obesity	Frontal expansive cerebral lesion
11	M	75	17	Mix	No	No	Yes	Yes	No	Epilepsy	Increase of cerebrospinal fluid space
12	M	65	57	Dep	No	No	Yes	No	Yes	-	-
13	M	67	47	Dep	No	No	Yes	Yes	No	Diabetes	Increase of cerebrospinal fluid space
14	M	73	33	Man	No	No	Yes	Yes	No	-	-
15	M	71	28	Dep	No	No	Yes	No	No	Diabetes	Chronic vascular ischemia
16	M	79	79	Man	Yes	Yes	No	No	No	Ischemic cardiopathy	-
17	M	78	74	Dep	No	Yes	No	Yes	No	Hypertension	Chronic vascular ischemia
18	F	69	68	Man	No	No	Yes	Yes	No	Hypertension	Chronic vascular ischemia
19	F	88	88	Man	Yes	Yes	Yes	Yes	No	Hypertension	Chronic vascular ischemia
20	F	65	20	Dep	No	No	Yes	Yes	No	Hypertension	-

General medical conditions were found in the majority of patients (N = 14), and were mainly represented by chronic cardiovascular disorders. Patients suspected by medical staff of having organic mental disorders (N = 10) had undergone neuroradiological examination at the time of admission. All of the participants showed concomitant organic cerebral pathology: five displayed chronic vascular ischemia; two had enlarged cerebrospinal fluid spaces; one patient was suffering from cortical atrophy; one showed lacunar cerebral strokes; and one presented an expansive frontal lesion.

We also compared older patients in our sample with a sex-matched 20 control sample participants (< 65 years), who had a mean age of 34.6 years (± 11.7).

The older-younger patient comparison yielded an average mood disorder onset age that was significantly lower for the younger group (t = 4.0, p < .001). No significant differences were found in the rates of patients who showed irritability (according to criterion A of DSM IV for manic episode) and delusions during hospitalisation. A tendency towards a higher rate of hallucinations was found in the older group (χ^2 ^= 4.3, p = .09, FET). The younger patients showed a significantly higher percentage of family history of mood disorders (χ^2 ^= 4.0, p < .05) (Table 2).

**Table 2:** Demographic and clinical features: comparison between patients aged > or < 65 years

	Pts aged ≥ 65 (N = 20)		Pts aged < 65 (N = 20)			
	Mean	Sd	Mean	Sd	T/z	p

Age at onset	48.9	23.6	25.6	12.0	3.9	0.0004
	N	%	N	%	χ^2^	p
Irritability	12	60	12	60	0	1
Delusions	12	60.0	14	70.0	0.44	0.51
Hallucinations	6	30.0	1	5.0	4.33	0.09*
Family history for mood disorders (two missing values in both groups)	6	33.3	12	66.7	4.0	0.05

* two-tailed Fisher's exact test

## Discussion

Our study showed that manic cases represented 7% of the entire geriatric population admitted to our public psychiatric ward over a 5-year period. This percentage largely reflects literature findings on the "treated prevalence" rates of elderly mania [8,9]. The reported decrease of the prevalence of manic episodes in late life could be attributed to disorder-related factors (e.g. excessive mortality, recovery) and/or an interaction of these factors with aging and case-sampling (e.g. institutionalization, cohort differences); a progressive reduction of morbility with age has also been suggested [10].

The mean mood disorder onset age of the older adults in our sample was about 50 years of age – a finding similarly reported in other studies [11,12]. In particular, approximately half of our patients presented a mood disorder onset age that can be considered "typical" [13], and the remaining participants were "late onset" BD patients [14]. This finding is in line with the observation that BD in the elderly is a heterogeneous phenomenon, with a large proportion of patients making up a part of a population of "classic" bipolar individuals with acute episodes extending into their later years. It is more difficult to find a clear diagnosis of "vascular mania" [15], which might more clearly point to an etiological link with "organic" BD. Steffens and Krishnan [16] maintain that this subtype of elderly mania includes cognitive impairment, and a history of neurological signs and symptoms. Unfortunately, we would need more information in order to make such a retrospective diagnosis.

Several authors [14] have found that mania in the elderly is associated with neurological or systemic illnesses, or may even be precipitated by different types of medication. In fact, neuroimaging studies have yielded more signs of atrophy and cerebral vascular lesions in older bipolar patients than in age-matched unipolar patients and in control cases [6,15,17].

Unfortunately, only patients with positive neurological examination had undergone neuroimaging, although no patient had specifically been admitted due to neurological symptoms. However, six out of 10 of our older manic patients who underwent imaging showed signs of cerebral vascular lesions, reinforcing the issue that elderly mania is a heterogeneous phenomenon supporting other findings in the literature [18,19], i.e., of an association between elderly mania and cerebrovascular (especially right-hemisphere) lesions. It is difficult to clarify the pathogenetic role of the neurological damage – i.e., causative, or the direct outcome of bipolar patients' particular lifestyle, which can lead to cardiovascular risk factors (Type II diabetes, hypertension, smoking-linked factors, etc...), or merely a direct association. The possibility that cerebral damage might have unleashed or accompanied the manic episode, or even affected treatment management and prognosis in some of these cases cannot be excluded [17].

Our data revealed an overlapping in the presence of irritability and psychotic symptoms of both younger and elderly manic inpatients, partly corroborating the data trend in the literature [20]. Our results reflect findings from studies reporting more frequent family history of affective disorder in young patients [11,21], suggesting that a higher genetic burden may be associated with juvenile-onset BD. Accordingly, the later onset observed in older bipolar patient samples could be explained by a low genetic susceptibility for mood disorders that require more time to emerge, whether spontaneously or triggered by life events or medical and neurological comorbidity [22]. The generalizability of our results is limited by the paucity of the sample, which also limited the possibility of performing other comparisons (i.e. organic vs. non-organic, early onset vs. late onset mania). Another limitation is the retrospective design, which was conducted exclusively on the basis of medical records without standardized ratings. Moreover, the unavailability of prospective follow-up provided no information on patient outcomes, and multiple comparisons with a small sample could limit our conclusions.

## Conflict of interests

All authors had no conflict of interest – including financial or personal relationships with people or organizations that might inappropriately influence, or be perceived to influence the work at least three years before the present work was conducted.

## Authors' contributions

AB participated in the conception of the study, and in its design and coordination, performed the statistical analysis and drafted the first version of the manuscript. PS carried out the collection and management of data, managed the literature searches, and participated in the first drafting of the manuscript. FC carried out the collection and management of data, managed the literature searches, and participated in the first drafting of the manuscript. ML participated in the conception of the study, and in its design and coordination. LM participated in the conception of the study, and in its design and coordination. LL participated in the conception of the study, and in its design and coordination. GBC participated in the conception of the study, and in its design and coordination. All authors contributed to, and approved the final manuscript.

## Funding Sources

No funding was provided for this research.
